# Revisiting the psychometric properties of the McArthur admission experience survey: Validating the Portuguese version using a bifactor approach

**DOI:** 10.1016/j.heliyon.2024.e24114

**Published:** 2024-01-09

**Authors:** Deborah Oyine Aluh, Sofia Azeredo-Lopes, Barbara Pedrosa, Manuela Silva, Ugnė Grigaitė, Ana Rita Martins, Maria Ferreira de Almeida Mousinho, Graça Cardoso, José Miguel Caldas-de-Almeida

**Affiliations:** a Lisbon Institute of Global Mental Health, Lisbon, Portugal; b Comprehensive Health Research Centre (chrc), NOVA Medical School, NOVA University of Lisbon, Lisbon, Portugal; c Department of Clinical Pharmacy and Pharmacy Management, University of Nigeria Nsukka, Nigeria; d Department of Statistics and Operational Research, Faculdade de Ciências, Universidade de Lisboa, Portugal; e Centro Hospitalar De Vila Nova De Gaia/Espinho, E.P.E.| V. N. Gaia/Espinho Hospital Centre, Portugal; f Department of Mental Health Unidade Local de Saúde do Baixo Alentejo, Beja, Portugal

**Keywords:** McArthur admission experience survey, Perceived coercion, Psychometric, Validation, Portuguese, Bifactor model

## Abstract

Cultural factors play a significant role in shaping the perception of coercion during psychiatric admissions. The present study aimed to assess the psychometric properties of the Portuguese Admission Experience Survey(P-AES).

The study employed a cross-sectional approach in five psychiatric departments in three regions of Portugal. A total of 208 patients participated in the survey. Reliability was assessed through internal consistency and test-retest procedures. Internal validity was analyzed using a two-parameter logistic item response model, exploring three models, including a bifactor model. Convergent validity was determined by correlating AES scores with the Coercion Ladder (CL), Client Assessment of Satisfaction (CAT), and Global Assessment of Functioning (GAF) scale. Discriminatory power was assessed by comparing scores between patients with voluntary and involuntary admission status.

The P-AES demonstrated satisfactory internal consistency and test-retest reliability. The bifactor model exhibited superior fit compared to the one-factor and three-factor models. Correlations between P-AES and CL, as well as CAT scores, indicated good convergent validity. Additionally, P-AES scores were notably higher in patients with compulsory psychiatric hospital admission compared to those admitted voluntarily, confirming its discriminatory power.

The bifactor model suggests that all three domains of the AES should be used to measure the subjective experience of coercion.

## 1 Introduction

The significant attention given to the use of coercion in mental health care has prompted a global movement to minimize its use. Coercion encompasses both actions performed upon someone and the subjective experience resulting from those actions. So, it represents both objectively observable actions and subjectively perceived outcomes [1]. Perceived coercion is the subjective experience of coercion. It has been suggested that the power dynamics between patients and care providers may play a more significant role in shaping the perception of coercion than the actual coercive acts themselves [2].

There is a growing interest in perceived coercion, as it has been found to have significant detrimental effects on patients' prognoses [3]. It is not only related to legal status or the experience of coercive practices but also the level of patient involvement in treatment decisions and knowledge of legal issues [4]. High levels of perceived coercion can damage the therapeutic relationship with care providers [5], while lower levels are linked to higher satisfaction with treatment [6]. Several tools have been developed to measure the subjective experience of coercion by patients of psychiatric services, such as the Cantril Ladder [[7], [8], [9]], Coercion Experience Scale [[10], [11], [12], [13], [14], [15]], the Experienced Coercion Scale [16], and Nordic Admission Interview [8,17]. Nevertheless, the Admission Experience Survey (AES), designed for the MacArthur Coercion Study, stands out as one of most commonly used tool [7,[17], [18], [19], [20], [21], [22], [23], [24], [25], [26], [27], [28]].

The AES is a questionnaire consisting of 16 items based on the MacArthur Admission Experience Interview. It aims to examine the experience of hospitalization, patients' interactions with clinical staff and family members during admission, as well as their input in the decision to be admitted [23]. The experiences are categorized into three groups: (i) feelings of coercion or perceived coercion, (ii) negative pressures, and (iii) voice or procedural justice. The Perceived Coercion domain centers around concepts such as freedom, choice, initiative, control, and influence regarding admission to the hospital [29,30]. The Negative Pressure domain pertains to experiences of being forced, threatened, or physically compelled to enter the hospital [31]. Lastly, the Voice or Procedural justice domain relates to having an opportunity to express one's opinion regarding hospital admission [23,32]. The AES has demonstrated favorable psychometric properties and has been valuable in assessing the subjective experience of coercion in various psychiatric clinical environments [33,34]. Despite facing criticism for its focus solely on the hospital admission process and its limited ability to assess the impact of other coercive measures in the same situation [10], the AES still holds significant value because the admission experience plays a crucial role in shaping the overall impact of other coercive measures throughout admission and therapeutic relationships formed [[35], [36], [37]]. The perception of coercion during psychiatric admission can be influenced by context, culture, and beliefs [31,38]. Hence, it is crucial to examine the impact of diverse cultures on the perception of coercion during psychiatric admissions. Research in this area among Portuguese speaking populations seems to be hindered by the absence of a culturally adapted and standardized tool. Validating the Portuguese version of the Admission Experience Survey will facilitate meaningful comparisons of findings across cultures and pave the way for further investigation into factors that may influence the admission experience.

Psychometric analysis is of utmost importance for ensuring the instrument's reliability, validity, and appropriateness for its intended purpose. It allows for the verification of how well the included items align with the underlying theoretical framework of the instrument, facilitating the evaluation of the phenomenon of interest. These attributes contribute to the overall quality and credibility of research and provide a solid foundation for evidence-based interventions. Not many studies that have used the McArthur Admission Experience survey have validated the instrument in their context before use. The validation processes of the Swedish [26] Chinese [31], French [33] and Italian [34] versions of the AES have been reported. However, doubts have arisen regarding the actual factor structure of the AES due to concerns about the methodology used in the previous psychometric analyses. To ensure accurate assessment of internal validity, the two-parameter logistic (2 PL) item response models are preferred considering the dichotomous nature of the AES. This method was solely employed in the validation study of the French version of the AES. Earlier investigations have indicated a three-factor structure, and the French study also supports the same three-factor structure. However, the French study goes on to report that the one-factor structure also exhibits good fit properties. A bifactor model is recommended to explore complex constructs that consist of moderately related dimensions [39]. It allows for investigating whether there is a general factor present, and to what extent each specific dimension is distinct from this general factor [39]. Thus, it seems reasonable to hypothesize that the AES may be comprised of a general factor (i.e., subjective experience of coercion during admission) that accounts for the commonality shared by its dimensions.

Building upon previous studies, this article proposes a bifactor structure for examining the subjective experience of coercion during admission. Notably, as no previous research has explored the applicability of a bifactor model for the subjective experience of coercion during admission, the findings from this study will have significant implications for further investigations of the concept. The present study aimed to evaluate the validity and reliability of the Portuguese version of the AES, and to assess whether the bifactor model gives a better fit than the three-factor model or one-factor model.

## 2 Materials and methods

### 2.1 Study design and setting

The research employed a cross-sectional approach and took place in five selected public psychiatric departments located in Lisbon, Porto, and Alentejo representing districts from north and south of Portugal between February 2022 and May 2023. The study sample included patients on admission in the selected psychiatric departments who met the eligibility criteria of being at least 18 years old and being able to give informed consent to participate in the study. Patients with dementia were excluded. Ethical approval was granted by the Ethics committee of Nova medical school (100/2021/CEFCM) and the ethics committee of each psychiatric department.

### 2.2 Measures

The MacArthur Admission Experience survey was used to enable rapid assessment of patients' perceptions of psychiatric hospital admission [33]. The Perceived Coercion domain has a scoring range of 0–5 while the Negative Pressure domain has a scoring range from 0 to 6. The Voice domain has a scoring range from 0 to 3. Item 9 was discarded as in the original scale, and the 16th item, which asks, “How did being admitted to the hospital make you feel?” and gives four possible options, was not included in the scoring. The GAF (Global Assessment of Functioning) is a scale utilized to assess an individual's social, occupational, and psychological functioning [40]. It ranges from 1 corresponding to severely impaired to 100 corresponding to extremely high functioning and is based on the DSM-IV (Diagnostic and Statistical Manual of Mental Disorders, Fourth Edition). The Coercion Ladder (CL) is a visual analogue tool that asks the patient to mark the level of their perceived coercion on a scale of 1 (indicating the minimum use of coercion – “I came totally on my own will and initiative”) to 10 (indicating the maximum use of coercion) [41]. The Client's Assessment of Treatment Scale (CAT) is a patient-reported outcome measure consisting of seven items designed to assess the patient's appraisal of in-patient care [42]. These items explore the patient's satisfaction with the treatment received, the treating clinicians, other mental health professionals, medications, other received treatments, and the overall care received. Each item is rated on a 10-point Likert scale ranging from 0 ("not at all") to 10 ("yes entirely").

### 2.3 Procedure

The McArthur Admission Experience Survey was translated into Portuguese by a bilingual native speaker with clinical experience and subsequently reviewed by a team of experts who were also native speakers. Then the translated questionnaire underwent back-translation from Portuguese to English by an independent translator and was checked again by the team of experts for accuracy. The instrument was then piloted among five patients admitted to one of the psychiatric departments selected for the study to assess for any difficulties or misunderstandings related to the terminology used. No changes were required at this stage. The study instrument, consisting of the Portuguese-AES (P-AES), CL, and CAT, was administered to the participants as soon as they were stable within the first seven days after admission (T0). The same instrument was administered to the respondents a second time before discharge (T1). Sociodemographic information (such as gender, age, employment status, marital status, nationality, the legal status of admission, and previous involuntary admissions) was obtained from the patients' records. Additionally, GAF scores and diagnoses according to ICD-10 criteria were recorded at both T0 and T1. The reliability of the P-AES scores was assessed using a test–retest approach as the instrument was administered at two different times. The average length of time for the second assessment was 12.54 (SD: 10.37) days. Validity was estimated based on the first assessment, by testing the three-factor model (perceived coercion, negative pressure, and voice subdomains) as reported in previous studies [18,33,34], a one-factor model [33], and a new bifactor model. To assess convergent validity, the following hypotheses were proposed: Firstly, the P-AES, including its perceived coercion and negative pressure domains, would exhibit positive correlations with the Coercion Ladder. Conversely, the Voice domain would demonstrate a negative correlation with the Coercion Ladder. Secondly, it was anticipated that the P-AES, perceived coercion domain, and negative pressure domain would display negative correlations with the GAF and CAT, while the Voice domain would show positive correlations with the GAF and CAT. This was based on the idea that individuals with higher functioning might experience less coercion during the admission process [33], and those who are more satisfied with their treatment could perceive lower levels of coercion. To evaluate discriminatory power, the AES's ability to distinguish between involuntarily admitted and voluntarily admitted patients was examined.

### 2.4 Statistical analysis

The data's normality was assessed using the Kolmogorov-Smirnov test, and a non-normal distribution was indicated. Consequently, non-parametric statistical methods were employed, namely the Wilcoxon Signed Rank test, for comparing paired data. To estimate the reliability of the MacArthur Admission Experience survey and its domains, McDonald's model-based Omega (ω) [43] and Cronbach's alpha coefficient were employed. Both these coefficients were obtained through the *psych* R package [44]. The test–retest reliability was assessed using Spearman's rank correlation and intraclass correlation coefficient (ICC). The cutoff criteria, based on Ko and Li's approach, were applied. If the ICC is below 0.50, it points to unreliable results. A value between 0.50 and 0.75 suggests a moderately reliable outcome, while a range of 0.75–0.90 indicates good reliability. Anything above 0.90 signifies excellent reliability [45]. The presence of significant changes in P-AES scores between the first and second assessments was evaluated using Wilcoxon tests.

To evaluate the reliability of the items, a two-parameter logistic (2 PL) item response model was utilized, given that the items were dichotomous. Item response theory (IRT) evaluates the validity of measurement scales by elucidating the connection between a latent trait (in this case, perceived coercion), the characteristics of the items within the scale, and how respondents answered each item individually [46]. This method estimates both item discrimination and difficulty. Additionally, a Confirmatory Factor Analysis (CFA) was conducted using maximum likelihood estimation to assess the adequacy of the factor structure of the P-AES. Three models were estimated: a three-factor model incorporating perceived coercion, negative pressure, and voice domains; a single-factor model with a general perceived coercion domain; and a Bifactor model, assuming all items load into a general domain, with subsets of items also loading into specific domains [47]. The models were obtained by fitting maximum likelihood factor analysis to dichotomous data under the item response theory paradigm using Cai's [48,49] Metropolis-Hastings Robbins-Monro (MHRM) algorithm and quasi-Monte Carlo integration for the parameters estimates. To evaluate the model fit, we considered several indicators, including the root mean square error of approximation (RMSEA), the comparison fit index (CFI), and the Tucker-Lewis fit index (TLI). A RMSEA value of ≤0.06, and CFI and TLI values of ≥0.95 indicated that the model fit was good. Alternatively, RMSEA values of ≤0.08, and CFI and TLI values of ≥0.90 were deemed acceptable [50]. Bifactor models are susceptible to overfitting [51,52], leading to a biased preference for these models when using model selection indices. To prevent excessive model fitting, four commonly employed model selection indices were used to compare the model fitting. These indices include Akaike's information criterion (AIC), Bayesian information criterion (BIC), Hannan–Quinn criterion index (HQ), and sample size-adjusted BIC (SABIC) [[53], [54], [55], [56]]. The computation of these indices aimed to ensure a reasonable and unbiased evaluation of the models.

To evaluate the convergent validity of the P-AES with other scales, Spearman correlation coefficients were calculated. Since the convergent validity coefficients do not have established criteria, values exceeding .30 were deemed satisfactory, corresponding to a medium effect size according to Cohen [57]. To determine the discriminatory power of the P-AES, an independent sample student t-test was conducted to compare the mean scores of patients admitted voluntarily and involuntarily. All statistical analyses were conducted using R software [58] with its *mirt* package being used to fit the Item Response Theory (IRT) models [59]. Two-tailed statistical tests were utilized, and the significance level was established at α = 0.05.

## 3 Results

### 3.1 Characteristics of study participants

A total of 208 patients from the five psychiatric departments were recruited to complete the survey. The required socio-demographic and clinical information was completed for 152 patients. The mean age of the sample was 46.53 (SD = 16.85), and more than half (54.61 %, n = 83) of them were female. A significant proportion of the participants were not employed (48.68 %, n = 74), and approximately one-fifth of them were retired (22.37 %, n = 34). Most of the participants were single (53.95 %, n = 82), and a small portion of them were immigrants in Portugal (12.5 %, n = 19). Additionally, about one-third of the sample had an involuntary admission legal status (32.24 %, n = 49). The most prevalent diagnosis among the participants were psychotic disorders (44.08 %, n = 67).

### 3.2 Psychometric properties of the P-AES

The measures of internal consistency and test-retest reliability were found to be satisfactory, except for the Voice domain which has a Cronbach alpha of 0.654. The intraclass coefficients showed moderate levels of reliability overall, except the Voice domain, which exhibited poor reliability. Significant changes were observed between the scores obtained in the first and second assessments (p < 0.05) (Table 1).

**Table 1:** Reliability of the P-AES.

	Internal consistency		Test-retest reliability		Wilcoxon test
Domains	**McDonald's ω**	**Cronbach's α**	**Spearman rho**	**ICC (2,1) 95 % CI**	**V (p-value)**
Perceived Coercion	0.850	0.809	0.701**	0.703 (0.591–0.747)	3167 (0.04)
Negative Pressures	0.853	0.756	0.526**	0.534 (0.41–0.636)	8136 (<0.001)
Voice	0.727	0.654	0.331**	0.286 (0.155–0.408)	8195 (<0.001)
Total Scale	0.910	0.880	0.6113**	0.539 (0.183–0.436)	7718 (<0.001)

*p < 0.05. **p < 0.01.

Factor loadings ranged from 0.483 to 0.994 for the one-factor model, from 0.582 to 0.989 for the three-factor model, 0.392 to −0.951 in the general factor of the bifactor model, and 0.0506 to 0.851 for the specific factors of the bifactor model. The bifactor model had the best-fit properties compared to the one-factor model and the three-factor models (Table 2). The four model selection indices for the bifactor model were consistently lower than those of the other two models, with the three-factor model showing the highest values (Table 3).

**Table 2:** Standardized loadings of the three models.

Item*	One-factor model		Three-factor model				Bifactor model				
	**F1**	**h2**	**F1**	**F2**	**F3**	**h2**	**F1**	**f1**	**f2**	**f3**	**h2**
1	0.820	0.672	0.732			0.536	0.768	0.208			0.633
2	0.806	0.649		0.755		0.570	0.813		0.186		0.696
3	−0.929	0.864			0.987	0.975	−0.951			0.051	0.907
4	0.994	0.989	0.989			0.978	0.933	0.356			0.998
5	−0.750	0.563			0.766	0.586	−0.801			0.449	0.843
6	−0.647	0.419		0.926		0.858	0.640		0.583		0.750
7	0.928	0.862	0.983			0.966	0.792	0.574			0.957
8	0.680	0.463		0.789		0.623	0.684		0.370		0.605
10	0.483	0.234		0.771		0.595	0.392		0.851		0.878
11	0.512	0.262		0.685		0.470	0.470		0.543		0.516
12	0.610	0.372		0.582		0.338	0.591		0.178		0.381
13	−0.657	0.432			0.593	0.352	−0.713			−0.505	0.763
14	0.513	0.263	0.651			0.424	0.456	0.471			0.429
15	0.633	0.401	0.657			0.431	0.571	0.394			0.481

h2 = communality of the item.

**Table 3:** Comparisons of model fit.

Model	χ^2^	df	*p*-value	RMSEA	90 % C.I. for RMSEA	CFI	TLI
One-factor model	205	77	<0.001	0.118	0.094–0.140	0.913	0.897
Three-factor model	282	77	0	0.149	0.126–0.171	0.860	0.835
2 PL Bifactor model	91.2	63	0.012	0.061	0.020–0.092	0.972	0.981
Direct comparison between the bifactor models with other models							
	**AIC**	**SABIC**	**HQ**	**BIC**	**logLik**	**χ2**	**p**
One factor model	1804	1794	1836	1883	−874	68.786	0
Three factor model	1908	1898	1940	1987	−926	172.934	0
Bifactor model	1764	1748	1811	1881	−840		

*χ2 = Chi-Square statistic; df = degrees of freedom; CFI = Comparative Fit Index; TLI = Tucker Lewis Index; RMSEA = Root Mean Square Error of Approximation, C.I. Confidence Interval.

As a supplementary addition to the factor loadings, the discrimination and difficulty of each item for the three models is presented using the Item Response Theory metric (Table 4).

**Table 4:** IRT parameterization for the P-AES.

Items	One-factor Model		Three-factor Model		Bifactor model (with 3 sub-factors)		
	Item difficulty	Item discrimination	Item difficulty	Item discrimination	Item difficulty	Item discrimination_(g)_	Item discrimination_(s)_
Item 1	−0.445	2.448	−0.375	2.070	−0.456	2.157	0.584
Item 2	0.110	2.340	0.104	1.915	0.107	2.512	0.575
Item 3	0.972	−4.410	4.498	13.424	1.233	−5.312	0.283
Item 4	−0.075	15.288	−0.037	9.646	−0.799	33.732	12.850
Item 5	0.611	−1.938	0.833	2.057	0.995	−3.436	1.926
Item 6	−1.533	1.468	−2.944	3.968	−2.393	2.177	1.985
Item 7	0.962	4.232	1.660	7.139	1.680	6.508	4.711
Item 8	−1.128	1.599	−1.311	2.135	−1.342	1.850	1.002
Item 10	−1.018	0.946	−1.415	2.074	−2.657	1.910	4.149
Item 11	−0.470	1.029	−0.545	1.619	−0.603	1.151	1.330
Item 12	0.333	1.318	0.341	1.224	0.334	1.279	0.384
Item 13	0.349	−1.518	0.439	1.289	0.554	−2.496	−1.767
Item 14	−0.468	1.015	−0.527	1.601	−0.555	1.028	1.060
Item 15	0.435	1.404	0.499	1.626	0.459	1.350	0.930

Correlation coefficients were in the expected direction and significant for all the variables tested except the associations with the GAF which were neither significant nor in the expected direction. Differences in the scores P-AES and its subscales were significantly different between voluntary and involuntarily admitted patients p < 0.05 (Table 5).

**Table 5:** Convergent validity and discriminatory power of the P-AES scores.

Convergent validity											
		Coercion Ladder	p-value		GAF		p-value		CAT		p-value
Perceived coercion subscale		0.401	<0.001		0.089		0.200		−0.260		<0.001
Negative pressure subscale		0.475	<0.001		0.117		0.100		−0.342		<0.001
Voice subscale		−0.450	<0.001		−0.126		0.090		0.339		<0.001
Total scale		0.441	<0.001		0.115		0.100		−0.299		<0.001
**Discriminatory power**											
	**Voluntary admission**			**Involuntary admission**							
	Mean score (SD)			Mean score (SD)		2-Sample *t*-test		df		p-value	
Perceived coercion subscale	2.136 (1.807)			3.250 (1.643)		−4.2		125		<0.001	
Negative pressure subscale	1.750 (1.574)			3.183 (1.927)		−5		96		<0.001	
Voice subscale	1.803 (1.1151)			1.067 (0.9181)		4.8		137		<0.001	
Total scale	5.500 (2.099)			7.067 (2.246)		−4.6		107		<0.001	

## 4 Discussion

The main objectives of this study were twofold: first, to evaluate the psychometric properties of the Portuguese version of the Admission Experience Survey (P-AES), and second, to investigate the factor structure of the P-AES in a sample of patients admitted to five psychiatric wards across three regions of Portugal. Reliability refers to the extent of consistency in the observed scores on a specific test for a particular group of individuals [45]. Test-retest reliability allows for measurement of stability over time, internal consistency measures homogeneity across items instantaneously, while inter-rater reliability allows for measuring equivalence by comparison across different observers. Validity, which refers to how accurately the items in the questionnaire measure a concept, is often linked to reliability as well [60]. To ensure the reliability of test scores, it is advised to gather, combine, and incorporate various sources of evidence [61]. In the case of the P-AES, satisfactory levels of internal consistency were observed based on both Cronbach's alpha and McDonald's omega, except for the voice domain, which showed marginally acceptable values. This might be because there are only three items on the voice subscale. When the number of test items is limited, it can violate the tau-equivalent assumption of the Cronbach alpha and lead to an underestimation of reliability [62].

Intraclass Correlation Coefficient (ICC) is a reliability measure that assesses both the correlation and agreement between measurements [45]. In this regard, the values were acceptable for the full scale and domains, except for the voice domain which had lower values than acceptable. The lower reliability observed in the voice domain highlights the greater risk of type 2 error in measurements of perceived procedural justice [63,64]. The low ICC observed for the voice domain has implications for the adjustment of sample size and power calculations, more subjects are required for a given effect size to be statistically significant [[65], [66], [67]]. Significant changes were observed in the total P-AES and its domains over time. Two main factors influence the evaluation of test-retest reliability: the changing nature of the construct being measured over time and the length of the time interval between the two tests [68]. The changes observed in this study may be attributed to the variable length of time for the second assessment which was as long as 2 months in some cases. In test-retest reliability, it is assumed that the true score being measured remains consistent over a brief period [69]. It may also reflect changes in functioning and experiences during stay in the ward. Certain psychological phenomena, such as mood or perceived coercion in this context, can undergo rapid changes in a short period. Furthermore, the interpretation of test-retest reliability assumes that the two test administrations are identical and independent, and that individuals' performance remains consistent across different time points [70]. In real-life test settings, meeting these assumptions can be difficult.

In keeping with previous studies, associations were observed among the domains and the entire scale [21,33]. The perceived coercion domain was positively associated with the use of negative pressures such as threats and physical force and inversely related to the voice subscale which is the sense of procedural justice [4]. To confirm the internal validity of the P-AES, three models were evaluated, two of them having been previously discussed in earlier studies [18,21,33]. The French validation reported that while a single total coercion score sufficed, the comparison between one- and three-factor models implied that Perceived Coercion, Negative Pressures, and Voice should not be treated as identical and recommended adopting the three-factor solution [33]. In our study, the bifactor model consistently displayed the most favorable fit characteristics when compared to the one-factor and three-factor models using multiple model selection indices. The bifactor modeling approach assumes that the interrelationships among items are accounted for by both a general factor (a shared underlying variable) and specific group factors. The general factor, denoting the shared aspect of the subjective experience of coercion during admission, can be understood as the general perceived coercion, a broadly defined concept. On the other hand, the three group factors represent the distinct elements of the three domains and can be interpreted as domain-specific facets of the subjective experience of coercion, each with a more specific definition. The bifactor structure captures both the uniqueness and commonality of the subjective experience of coercion. Therefore, the observed bifactor model in this study indicates that perceived coercion, negative pressures, and voice share certain commonalities under the general subjective experience of coercion factor, while also having separate and unique components independent from the general factor. This aligns with Hodge et al.'s original concept of a core construct, where the experience of coercion is comprehensively described through other group latent factors, such as perceived fairness, relationships with individuals exerting pressure, and the aspect of voice or validation [1]. Based on this finding, it is recommended that when assessing the subjective experience of coercion, all three domains should be used, and the total score of the AES along with the scores of its domains should be reported.

There is some concern regarding Item #13 on the AES (My opinion about coming into the hospital didn't matter), as it exhibited negative item discrimination parameters in both the one-factor and bifactor models. This is problematic since it implies that respondents with higher levels of the underlying trait (perceived coercion) are less inclined to endorse a more severe response option [46]. Previous research suggests that reverse-worded items in mixed-worded scales may be problematic [71]. This can be especially so in negative-concord languages like Portuguese. The reversal of wording has been found ineffective in addressing bias and can even be counterproductive in some cases [72]. Researchers translating the instrument should reconsider reversing the wording of this item, especially in languages that use negative concord.

Correlations between the P-AES scores and its domains, with the Coercion Ladder and the CAT were largely in line with our hypotheses, suggesting that the P-AES is a valid measure of experience of coercion. The AES scale and its domains did not exhibit the anticipated associations with the GAF, and these associations were not statistically significant. This suggests that functioning, as assessed by the GAF, did not show any significant associations with perceived coercion. These results align with earlier studies reporting that perceived coercion is not strongly linked to the severity of psychiatric symptoms [37,73], but differ from the findings of the French validation study [33]. Additionally, the P-AES demonstrated good discriminatory power, as evidenced by significant mean score differences between voluntarily and involuntarily admitted patients, which supports our initial hypothesis.

### 4.1 Strengths and limitations

The validation study utilized the two-parameter logistic (2 PL) item response theory approach, which is deemed more suitable for the AES due to its dichotomous item format. An advantage of Item Response Theory over Classical Test Theory is that item parameter estimates remain unbiased and independent of the sample, even with unrepresentative samples [74]. Additionally, we extended the current understanding of the AES factor structure by exploring a bifactor model, a novel approach not previously explored in validation studies. While this study makes a significant contribution to the existing literature, further research is necessary to validate whether the bifactor model accurately represents the subjective experience of coercion during admission samples from other countries. Nevertheless, several methodological limitations need to be acknowledged. Firstly, convenience sampling was used, which could impact the generalizability of the findings. Secondly, to enhance accuracy, the second assessment for the test-retest should have been conducted within 48 h. Thirdly, reliance on self-report ratings for concurrent measures introduces the potential risk of artificially high correlations due to mono-method bias [75]. Moreover, it is essential to acknowledge that prior research has shown bifactor models to excel in terms of fit statistics compared to other models. Consequently, there is a chance that the bifactor model might have captured some irrelevant information and demonstrated a better fit due to an inclination to overfit the data. However, the study took steps to address this issue by using multiple model selection criteria to minimize potential biases.

## 5 Conclusion

The study introduces a bifactor structure to model the subjective experience of coercion during admission. While the domains of perceived coercion, negative pressure, and voice represent distinct concepts, they also share common elements as part of the broader concept of the subjective experience of coercion. This finding would be valuable in future research aimed at gaining a deeper understanding of the subjective experience of coercion. Furthermore, our findings affirm that the P-AES serves as a valid instrument for assessing the subjective experience of coercion during admission among individuals with mental health conditions in Portugal. Validating this questionnaire is a crucial initial step, enabling the testing of additional hypotheses and formulation of interventions to improve the admission experience. When combined with qualitative approaches like on-site observations and interviews, the P-AES can function as a tool for self-evaluation, encouraging more extensive conversations about optimal procedures during the admission process. It is expected to foster the advancement of future research endeavors in Portuguese-speaking countries and stimulate cross-cultural comparisons on this subject.

## Ethics declarations

• This study was reviewed and approved by ethics committee of Nova medical school, with the approval number: 100/2021/CEFCM.
• All participants provided informed consent to participate in the study.

## Data availability statement

The data analyzed during the current study are available from the corresponding author on reasonable request.

## Funding details

The study did not receive any specific funding. Deborah Oyine Aluh is a PhD student receiving the support of a PhD fellowship from “la Caixa” Foundation (LCF/BQ/DI20/11780013). Barbara Pedrosa and Ugnė Grigaitė are PhD students receiving financial support from the 10.13039/501100001871FCT – Fundação para a Ciência e a Tecnologia (UI/BD/151073/2021 and UI/BD/151072/2021)

## CRediT authorship contribution statement

**Deborah Oyine Aluh:** Conceptualization, Methodology, Project administration, Writing – original draft, Writing – review & editing. **Sofia Azeredo-Lopes:** Formal analysis, Software, Writing – review & editing. **Barbara Pedrosa:** Methodology, Writing – review & editing. **Manuela Silva:** Data curation, Writing – review & editing. **Ugnė Grigaitė:** Writing – review & editing. **Ana Rita Martins:** Data curation, Writing – review & editing. **Maria Ferreira de Almeida Mousinho:** Data curation, Writing – review & editing. **Graça Cardoso:** Supervision, Writing – review & editing. **José Miguel Caldas-de-Almeida:** Supervision, Writing – review & editing.

## Declaration of competing interest

The authors declare that they have no known competing financial interests or personal relationships that could have appeared to influence the work reported in this paper.
