# Supplementary material for: Revisiting the psychometric properties of the McArthur admission experience survey: Validating the Portuguese version using a bifactor approach
Source: Heliyon. 2024 Jan 9;10(2):e24114. doi: 10.1016/j.heliyon.2024.e24114 (PMC10827453; doi:10.1016/j.heliyon.2024.e24114)
Supplement: Multimedia component 1 [file mmc1.docx]

**Estudo de Coerção de MacArthur**

**Questionário de Experiência de Admissão: Versão Curta**

"Vou agora ler-lhe algumas afirmações sobre a sua vinda actual ao hospital. Por favor responda “verdadeiro” ou “falso” a cada afirmação. Procure responder a cada questão individualmente, independentemente de lhe parecem similares entre si.”

|  | **Verdadeiro** | **Falso** | **Não sei** |
| --- | --- | --- | --- |
| **1.** Senti-me livre para fazer o que queria em relação a vir ao hospital. | **[ ]** | **[ ]** | **[ ]** |
| **2.**As pessoas tentaram forçar-me a vir para o hospital. | **[ ]** | **[ ]** | **[ ]** |
| **3.**Tive possibilidade suficiente de dizer se queria vir para o hospital. | **[ ]** | **[ ]** | **[ ]** |
| **4.**Eu escolhi vir para o hospital. | **[ ]** | **[ ]** | **[ ]** |
| **5.** Pude dizer o que queria em relação a vir para o hospital. | **[ ]** | **[ ]** | **[ ]** |
| **6.**Alguém me ameaçou de forma a que eu viesse para o hospital. | **[ ]** | **[ ]** | **[ ]** |
| **7.** Foi minha ideia vir para o hospital. | **[ ]** | **[ ]** | **[ ]** |
| **8.** Alguém tentou fisicamente fazer-me vir para o hospital. | **[ ]** | **[ ]** | **[ ]** |
| **9.** Não me pareceu que alguém quisesse saber se eu queria vir para o hospital. | **[ ]** | **[ ]** | **[ ]** |
| **10.** Fui ameaçado(a) com internamento. | **[ ]** | **[ ]** | **[ ]** |
| **11.** Eles disseram que iriam fazer com que eu viesse para o hospital. | **[ ]** | **[ ]** | **[ ]** |
| **12.** Ninguém me tentou forçar a vir para o hospital. | **[ ]** | **[ ]** | **[ ]** |
| **13.** A minha opinião acerca de vir para o hospital não teve importância. | **[ ]** | **[ ]** | **[ ]** |
| **14.** Eu tive bastante controlo acerca de vir para o hospital. | **[ ]** | **[ ]** | **[ ]** |
| **15.**Eu tive maior influência do que qualquer outra pessoa acerca de vir para o hospital. | **[ ]** | **[ ]** | **[ ]** |
| **16.** Como se sentiu em relação ao facto de ter sido internado no hospital?  Sentiu-se: |  |  |  |
| **a.** Zangado. | **[ ]** | **[ ]** | **[ ]** |
| **b.**Triste. | **[ ]** | **[ ]** | **[ ]** |
| **c.** Satisfeito. | **[ ]** | **[ ]** | **[ ]** |
| **d.**Aliviado. | **[ ]** | **[ ]** | **[ ]** |
| **e.**Confuso. | **[ ]** | **[ ]** | **[ ]** |
| **f.**Assustado. | **[ ]** | **[ ]** | **[ ]** |
