# Supplementary material for: Revisiting the psychometric properties of the McArthur admission experience survey: Validating the Portuguese version using a bifactor approach
Source: Heliyon. 2024 Jan 9;10(2):e24114. doi: 10.1016/j.heliyon.2024.e24114 (PMC10827453; doi:10.1016/j.heliyon.2024.e24114)
Supplement: Multimedia component 2 [file mmc2.docx]

**The MacArthur Coercion Study**

**Admission Experience Survey: Short Form**

"I am now going to read you some statements about your coming into the hospital this time. Please answer either "TRUE" or "FALSE" to each statement. Try to answer each question individually, no matter how similar it may sound to another."

|  | **True** | **False** | **Don't Know** |
| --- | --- | --- | --- |
| **1.** I felt free to do what I wanted about coming into the hospital. | **[ ]** | **[ ]** | **[ ]** |
| **2.**People tried to force me to come into the hospital. | **[ ]** | **[ ]** | **[ ]** |
| **3.**I had enough of a chance to say whether I wanted to come into the hospital. | **[ ]** | **[ ]** | **[ ]** |
| **4.**I chose to come into the hospital. | **[ ]** | **[ ]** | **[ ]** |
| **5.** I got to say what I wanted about coming into the hospital. | **[ ]** | **[ ]** | **[ ]** |
| **6.**Someone threatened me to get me to come into the hospital. | **[ ]** | **[ ]** | **[ ]** |
| **7.** It was my idea to come into the hospital. | **[ ]** | **[ ]** | **[ ]** |
| **8.** Someone physically tried to make me come into the hospital. | **[ ]** | **[ ]** | **[ ]** |
| **9.** No one seemed to want to know whether I wanted to come into the hospital. | **[ ]** | **[ ]** | **[ ]** |
| **10.** I was threatened with commitment. | **[ ]** | **[ ]** | **[ ]** |
| **11.** They said they would make me come into the hospital. | **[ ]** | **[ ]** | **[ ]** |
| **12.** No one tried to force me to come into the hospital. | **[ ]** | **[ ]** | **[ ]** |
| **13.** My opinion about coming into the hospital didn't matter. | **[ ]** | **[ ]** | **[ ]** |
| **14.** I had a lot of control over whether I went into the hospital. | **[ ]** | **[ ]** | **[ ]** |
| **15.**I had more influence than anyone else on whether I came into the hospital. | **[ ]** | **[ ]** | **[ ]** |
| **16.** How did being admitted to the hospital make you feel?  Did it make you feel: |  |  |  |
| **a.** Angry. | **[ ]** | **[ ]** | **[ ]** |
| **b.**Sad. | **[ ]** | **[ ]** | **[ ]** |
| **c.** Pleased. | **[ ]** | **[ ]** | **[ ]** |
| **d.**Relieved. | **[ ]** | **[ ]** | **[ ]** |
| **e.**Confused. | **[ ]** | **[ ]** | **[ ]** |
| **f.**Frightened. | **[ ]** | **[ ]** | **[ ]** |

**Subscales of the MacArthur Admission Experience Survey (AES) -- Short Form 1**

*Item 9 of the AES-Short Form was eventually dropped from these scales.*

**I. Perceived Coercion Scale**

**The scale is thus 0-5, with each "True" = 0, and each "False" = 1. See Gardner et al (1993), p.316.**

**1.** I felt free to do what I wanted about coming into the hospital.
**4.**I chose to come into the hospital.
**7.**It was my idea to come into the hospital.
**14.**I had a lot of control over whether I went into the hospital.
**15.**I had more influence than anyone else on whether I came into the   hospital.

**II. Negative Pressures Scale**

**2.** People tried to force me to come into the hospital.
**6.** Someone threatened me to get me to come into the hospital.
**8.**Someone physically tried to make me come into the hospital.
**10.**I was threatened with commitment.
**11.** They said they would make me come into the hospital.
**12.** No one tried to force me to come into the hospital.[*reverse scored*]

**III. Voice Scale**

**The "voice" scale is an early version of what became the "procedural justice" scale (see Lidz et al, 1995).**

**3.** I had enough of a chance to say whether I wanted to come into the   hospital.
**5.** I got to say what I wanted about coming into the hospital.
**13.** My opinion about coming into the hospital didn't matter.[*reverse   scored*]

**IV. Affective Reactions to Hospitalization**

**16.**How did being admitted to the hospital make you feel? Did it make you feel:

**a.**Angry
**b.** Sad
**c.** Pleased
**d.** Relieved
**e.** Confused
**f.** Frightened
